# Supplementary material for: Quantification of clinically applicable stimulation parameters for precision near-organ neuromodulation of human splenic nerves
Source: Commun Biol. 2020 Oct 16;3:577. doi: 10.1038/s42003-020-01299-0 (PMC7568572; doi:10.1038/s42003-020-01299-0)
Supplement: Supplementary file 1 — Supplementary Information [file 42003_2020_1299_MOESM1_ESM.pdf]

Supplementary Information

Quantification of clinically applicable stimulation parameters for precision near-organ neuromodulation of human splenic nerves

Isha Gupta[1,*], Antonino M. Cassará[2], Matteo Donega[1], Ilya Tarotin[1], Jason A. Miranda[1], David M. Sokal[1], Sebastien Ouchouche[1], Wesley Dopson[1], Paul Matteucci[1], Esra Neufeld[2], Matthew A. Schiefer[3], Alison Rowles[4], Paul McGill[5], Justin Perkins[6], Nikola Dolezalova[7], Kourosh Saeb-Parsy[7], Niels Kuster[2,8], Refet Firat Yazicioglu[1], Jason Witherington[1], Daniel J. Chew[1,*]

[1]Galvani Bioelectronics, Gunnels Wood Road, Stevenage, SG1 2NY, UK
[2]Foundation for Research on Information Technologies in Society (IT'IS), Zeughausstrasse 43, 8004 Zurich, Switzerland
[3]SimNeurix, LLC, Gainesville, FL, USA
[4] Non-Clinical Safety, Research and Development, GlaxoSmithKline, Park Road, Ware, SG12 0DP
[5]Bioimaging, GlaxoSmithKline, Park Road, Ware, SG12 0DP
[6]The Royal Veterinary College, Hawkshead Lane, North Mymms, Hatfield, AL9 7TA, UK
[7]Department of Surgery, University of Cambridge, and NIHR Cambridge Biomedical Research Centre, Cambridge, CB2 0QQ, UK
[8]Swiss Federal Institute of Technology (ETH) Zurich, 8092 Zurich, Switzerland

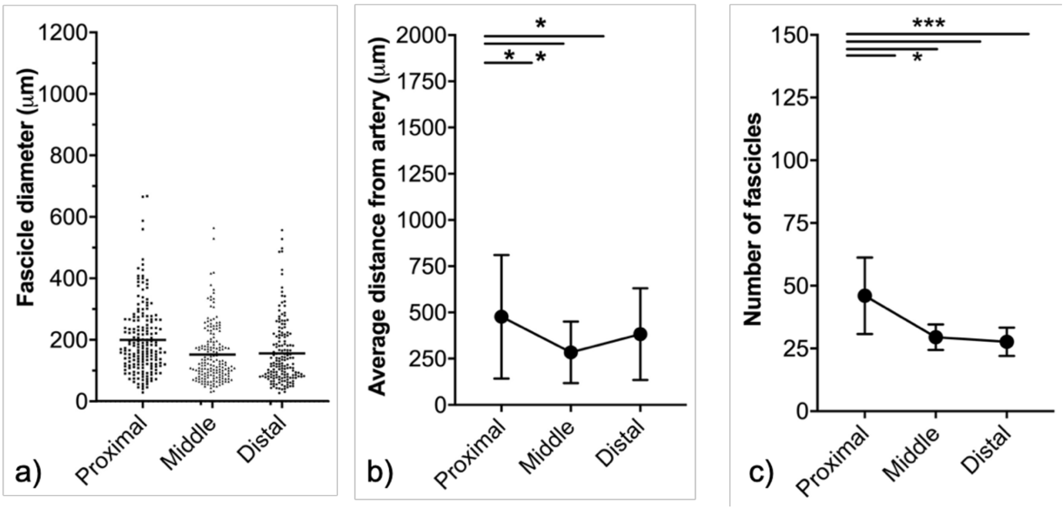

Supplementary Figure 1 Histomorphometric estimations from pig splenic artery. (a) Fascicle diameter, (b) average distance from the splenic artery, and c) Number of fascicles from the splenic artery in proximal, middle and distal region[1]. This is discussed in detail in the cited manuscript. In this manuscript only middle section has been used.

S.No.	Age	Sex	BMI	Blood type	Post-mortem Details	Tissue Quality
1	43	M	28	B+	~15 hours	• Good tissue morphology.<br>• Sequential H&E blocks of tissue were examined. The distribution of

						the fascicles depends on the location of the sample (proximal/middle/distal).
2	53	F	28	B+	~12.5 hours	• More nerve fascicles at the proximal and distal end. Less number of fascicles in the middle. Good preservation and morphology of artery and associated fascicles; some processing artefact of fascicles but not significant enough to limit downstream evaluation.
3	55	M	28	A+	~42 hours	• Some autolysis of tunica media and intima of artery wall. Tissue section of acceptable quality for interpretation.
4	46	F	30	O-	~20 hours	• Good preservation of artery and associated fascicles with little or no autolysis; some minimal processing artefact present in fascicles.
5	60	F	25	O+	~21 hours	• The tissue quality was of acceptable quality. Slight autolysis was noted in some sections, mainly in the lymphoid foci in the fat surrounding the artery and very slightly in the pancreas. The artery itself was well preserved and the nerves around it looked in good condition.
6	31	M	21	O+	Approx. 18 hours	• The tissue quality was generally good. Slight autolysis was noted in some sections, mainly in the lymphoid foci in the fat surrounding the artery and very slightly in the pancreas. The artery itself was well preserved and the nerves around it looked in good condition.

Supplementary Table 1 Demographics and Details of the human tissue used for histology for human model. Middle sections have been used in the manuscript for computational model. The other sections are discussed in detail in the referenced manuscript[1].

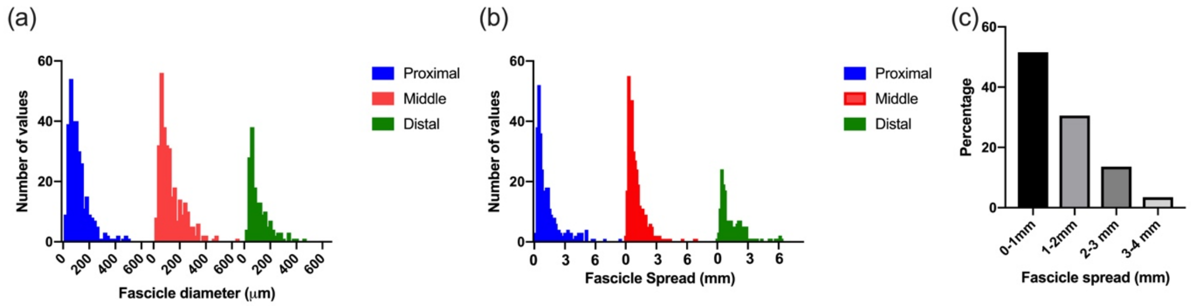

Supplementary Figure 2 Histomorphometric characterisation of human splenic anatomy. Splenic artery is divided into proximal (blue), middle (red) and distal region (green). (a) Fascicle diameter quantification. Diameter range in the middle section is in the range of 20-400μm approximately. (b) Fascicle diameter spread, and (c) Distribution of fascicles around the artery.

Sample Number	Lumen Diameter (mm)	Lumen Wall + Arterial wall (mm)	Accounting for shrinkage of the tissue (+10%) (mm)	Range of extravascular tissue (mm)	Total NVB (mm)
1	3.01	5.02	5.5	3.5	**9**
2	3.92	5.2	5.72	2.4	**8.12**
3	3.3	4.93	5.42	3.8	**9.2**
4	2.76	4.72	5.192	4.9	**10**
5	2.57	4	4.4	2.0	**6.4**

Supplementary Table 2 Histological estimated geometries of human splenic neurovascular bundles (~6mm to 10mm)

(a) (b)

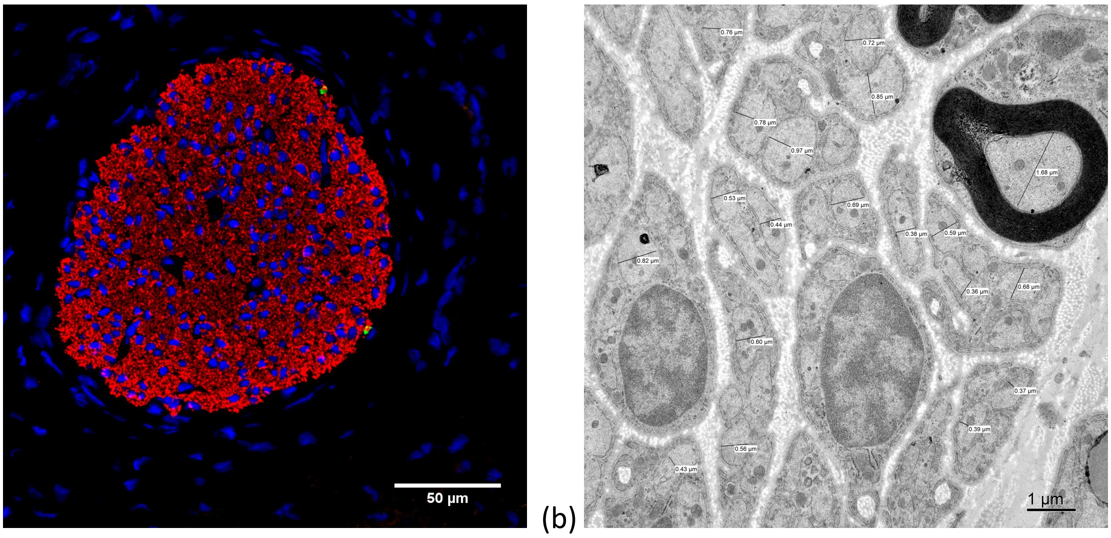

Supplementary Figure 3 (a) High magnification micrograph of a porcine splenic fascicle immunolabelled for NF-βTubIII (in red) and MBP (in green). Red: axons, Blue: Nuclei, Green: myelinated fibres. Scale bar = 50µm. The figures indicate the target site to be >99% unmyelinated[1]. **(b)** Transmission Electron microscopy image of axons within a nerve fascicle used to estimate axon diameter in humans. Diameter of axons are illustrated on the figure estimated in the range of 0.5-1µm.

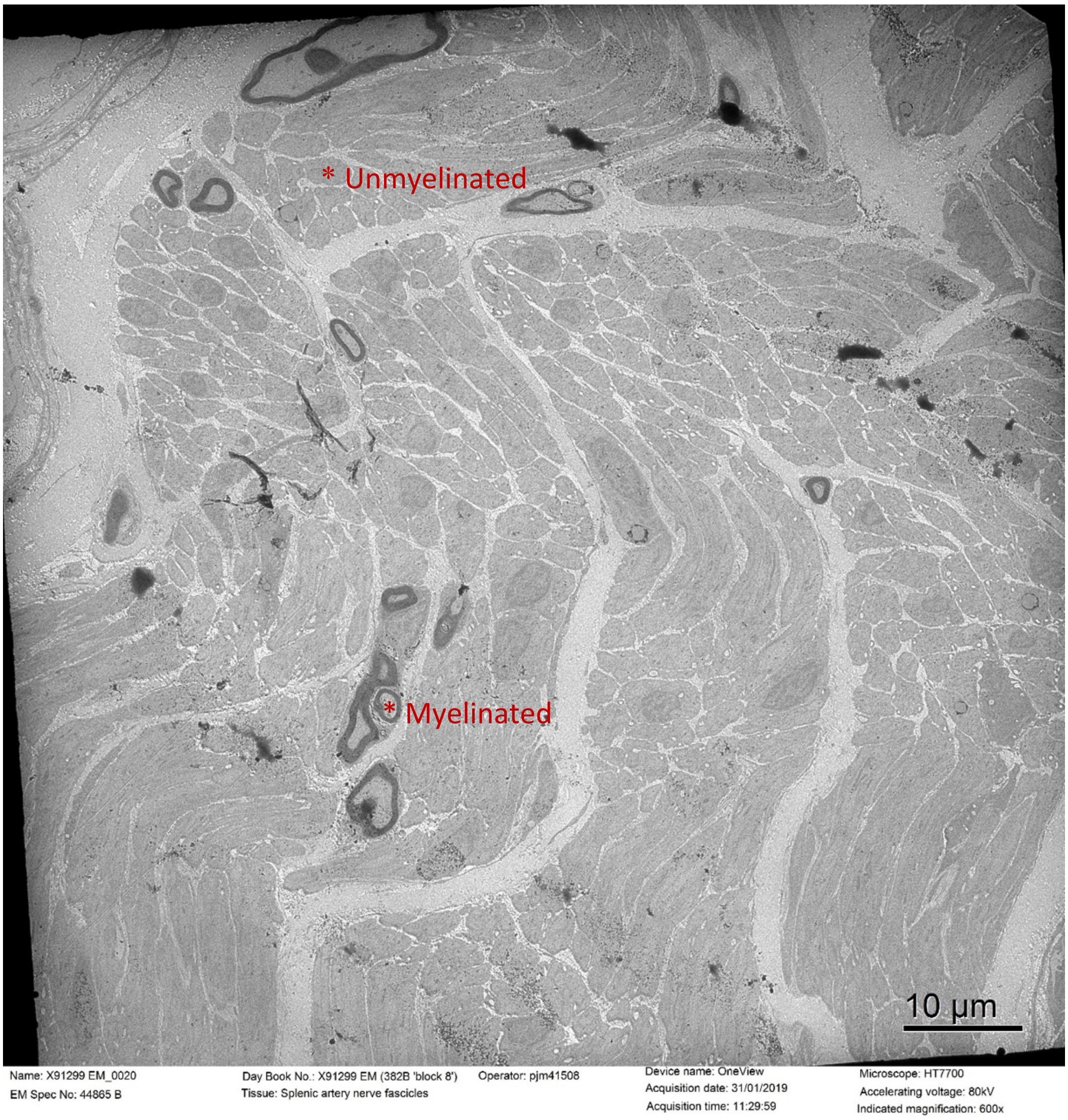

Supplementary Figure 4: A TEM image from large nerve fascicle. The image shows the target to be mainly unmyelinated.

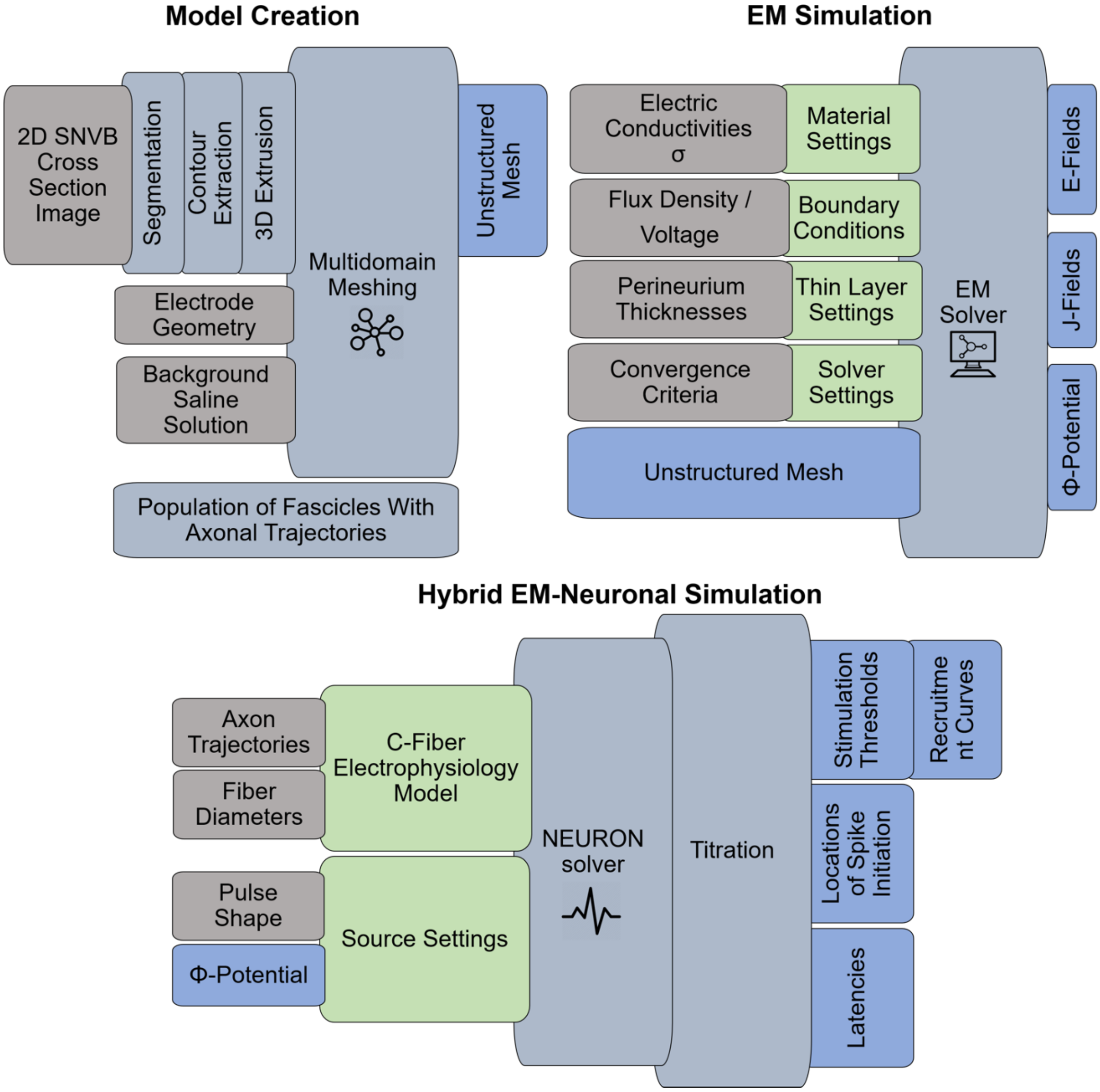

Supplementary Figure 5: *in silico* modelling pipeline. The pipeline can be divided into three principal components: Model Creation, EM Simulations, and Hybrid EM simulations. Grey: input parameters/data; Green: settings; Dark Blue: procedure; Light Blue: output data.

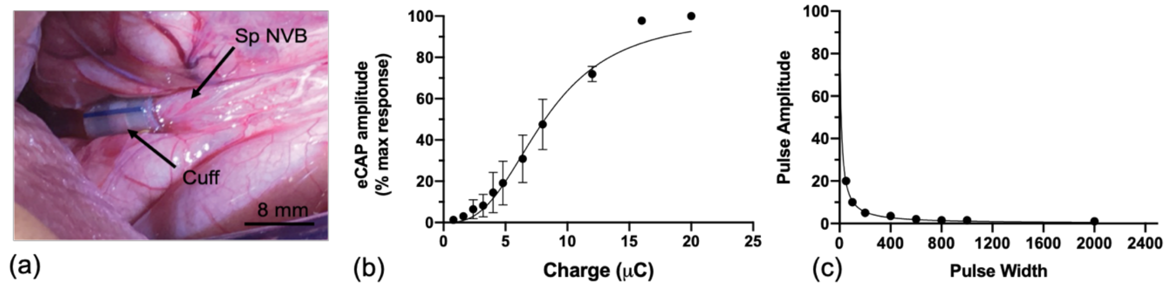

Supplementary Figure 6 (a) Peri-arterial cuff on porcine splenic neurovascular bundle, (b) Recruitment curve from pig splenic nerve when stimulated with 0.4ms pulse width at an increasing amplitude of 0-50mA in five animals. The errors bars represent the range. (c) Strength-duration relationship (black circles) of the splenic nerve obtained by stimulating the whole neurovascular bundle. Pulse width and pulse amplitude units are in μs and mA respectively. Threshold currents are defined as approximately 10% recruitment of the nerves. The range for recruiting 10% nerves in five different animals is approximately – 10,8,5, 15 and 8mA with a median of 8mA[1].

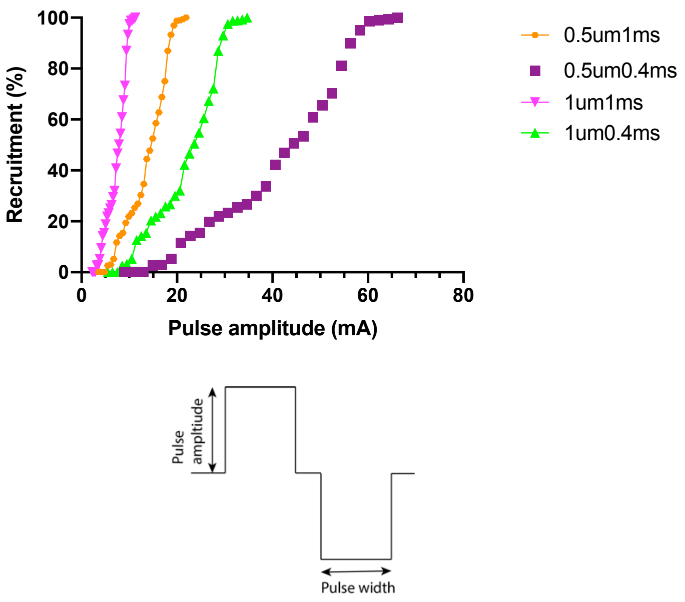

Supplementary Figure 7 *in silico* modelling results for different pulse widths in pig model. The graph illustrates recruitment vs pulse amplitude. The first part of the colour key denotes the axon diameter and second part shows the pulse width. Pulse amplitude thresholds for 1ms and 0.4ms are presented as follows:

	**Pulse width**	**1ms**	**0.4ms**
In silico pig	Thresholds [0.5-1] μm	3.6 -2.3mA	9 – 5.5mA
	Mean	2.95mA	7.25mA
	**Charge**	2.95μC	2.9μC
In vivo pig	**Median (from Supplementary Figure 3b)**		8mA
	**Charge**		3.2μC
	**% variation**		**9.3%**

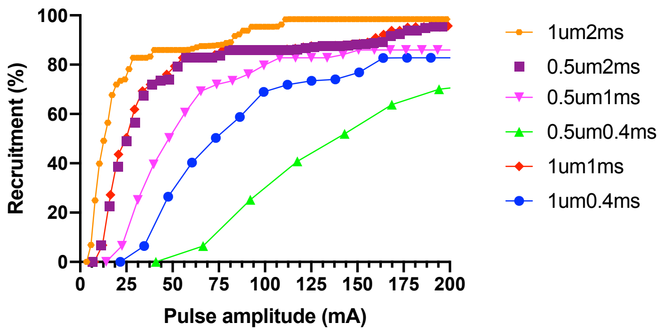

Supplementary Figure 8 *in silico* modelling results for different pulse durations in human model. The graph illustrates recruitment vs pulse amplitude. The first part of the colour key denotes the fibre diameter and second part shows the pulse width. Pulse amplitude thresholds for 2ms, 1ms and 0.4ms are presented as follows:

**Pulse width**	**2000µs**	**1000 µs**	**400 µs**
Thresholds [0.5 -1] µm	6.6-3.5 mA	14.1 -7.7mA	41 – 21.7mA

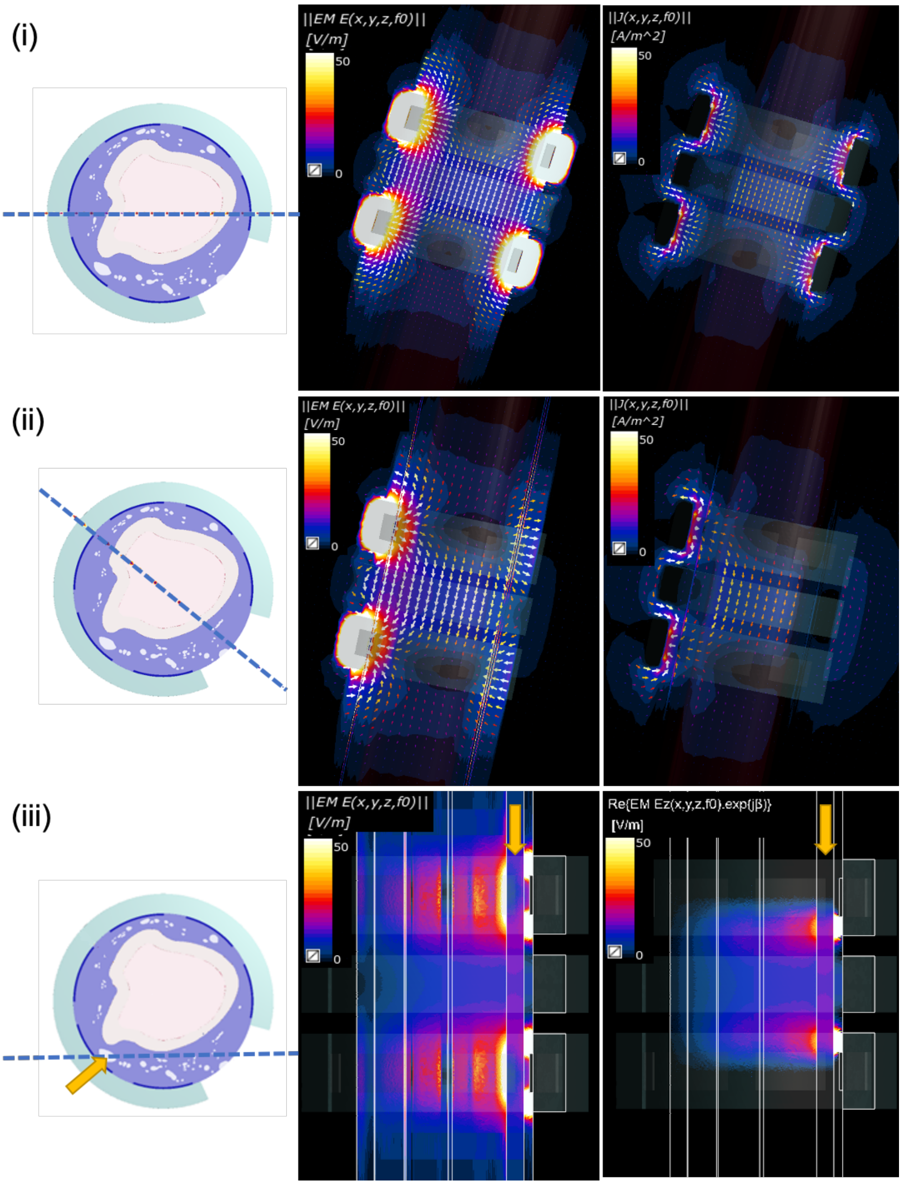

Supplementary Figure 9 (i) and (ii) E-field and current density field maps and vector plot on two longitudinal cross sections; one of which is cutting through cuff opening. (iii) E-field distribution in a slice crossing multiple fascicles (one indicated by the yellow arrow). The left image shows the total E-field. The right one is its longitudinal component relevant for neurostimulation.

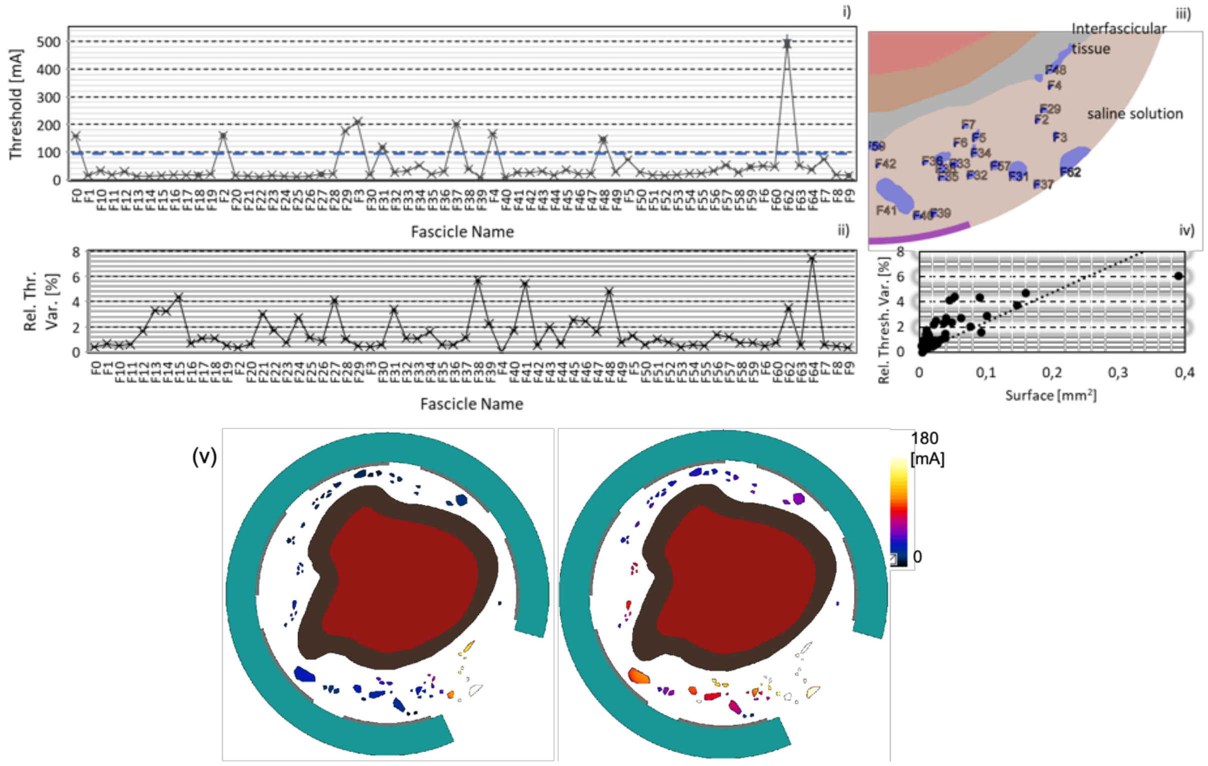

Supplementary Figure 10 Statistics about (i) stimulation thresholds and (ii) relative stimulation threshold variations within the fascicles in the human model. Image (iii) illustrates the bottom right side of the bundle with indication of fascicle's names. These are the fascicles present in the cuff opening. Purple colour represents one of the electrical contact pads. Plot (iv) illustrate the relation between relative variation of thresholds and fascicle's cross-section area. Comparing (i) and (iii) it can be observed that the fascicles having larger stimulation thresholds are those within the opening of the cuff. Threshold variations within the fascicles are below 8% with an average value of 1.5% and STD 1.5%. v) Stimulation threshold distributions within fascicles for the human model. Distributions are provided for two pulse durations: 1ms (left) and 0.4ms (right) for 1µm fibre diameter.

Sample	Age	Gender	BMI	Blood Group	Post Mortem Interval
HT1	63	F	23	O+	~36 hours
HT2	50	M	28	O+	~20 hours
HT3	40	F	32	O+	~30 hours
HT4	61	F	30	A+	~36 hours
HT5	48	F	23	O+	~24 hours
HT6	50	M	29	B-	~24 hours
HT7	55	F	22	O+	~13 hours
HT8	62	M	27	A+	~30 hours
HT9	42	F	36.5	O+	~24 hours

Supplementary Table 3 The table illustrates the list of human donor samples used for electrophysiology study.

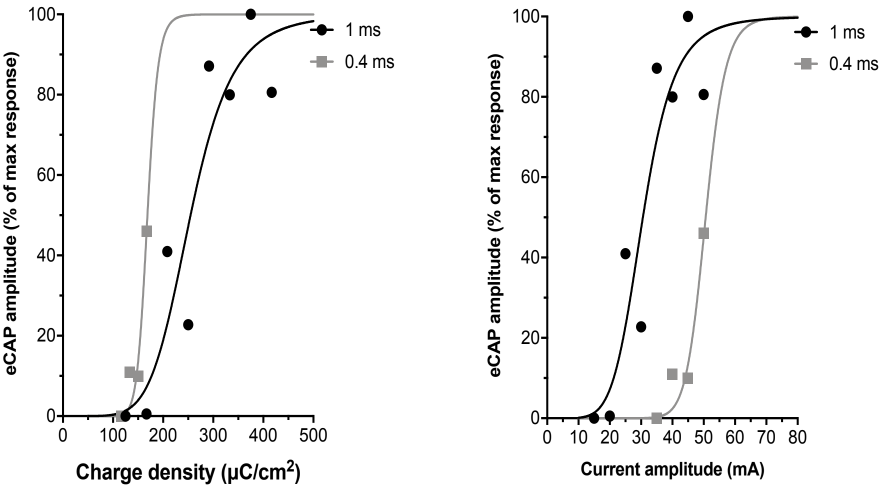

Supplementary Figure 11 Results from ex-vivo electrophysiological study in the human samples for an isolated fascicle. Charge density requirements (left) and estimated current amplitude (right).

	**Pulse width**	**2000μs**	**1000 μs**	**400 μs**
In silico human	Thresholds [1-0.5]μm	3.5-6.6mA	7.7-14.1mA	21.7-41mA
	Mean	5.05mA	10.9mA	31.35mA
	**Charge**	10.1μC	10.9μC	12.54μC
Ex vivo human Range	-	4-15mA	6-20mA	12.5-40mA
	Median	5.5mA	13mA	25mA
	**Charge**	11μC	13μC	10μC
Percentage variation	**In silico vs ex vivo**	**8.1%**	**16.15%**	**19.35%**

Supplementary Table 4 Comparative results from *ex vivo* human electrophysiology results and *in silico* modelling results.

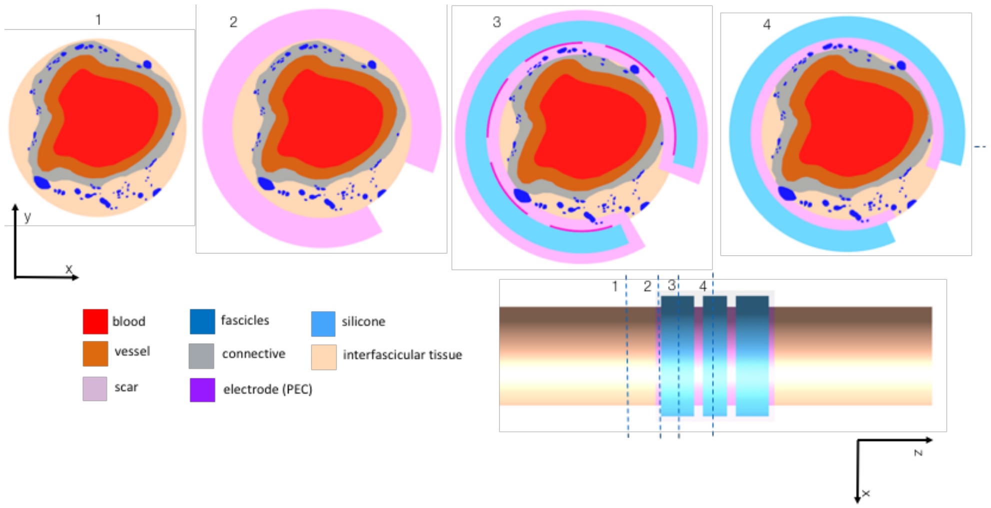

Supplementary Figure 12 Human computational model including scar tissue (encapsulation). The diameter from acute to chronic model has been kept constant. To accommodate scar tissue encapsulation; the existing 0.5mm of connective and adipose tissue was replaced by scar tissue. The position of fascicles/electrodes has been kept identical in both chronic and acute situation. Conductivity of the scar tissue was considered homogenous and isotropic.

References:

1. Donegà, M. *et al.* Human Relevant Near-Organ Neuromodulation of the Immune System via the Splenic Nerve. *Under Rev.*
