# Supplementary material for: Quantification of clinically applicable stimulation parameters for precision near-organ neuromodulation of human splenic nerves
Source: Commun Biol. 2020 Oct 16;3:577. doi: 10.1038/s42003-020-01299-0 (PMC7568572; doi:10.1038/s42003-020-01299-0)
Supplement: Supplementary file 2 — Description of Additional Supplementary Files [file 42003_2020_1299_MOESM2_ESM.pdf]

Description of Additional Supplementary Files

File Name: Supplementary Data 1
Description: Dataset for Figure 2n

File Name: Supplementary Data 2
Description: Graph Pad Prism File for Figure 2k, l, m

File Name: Supplementary Data 3
Description: Graph pad prism file for Figure 5

File Name: Supplementary Data 4
Description: Graph pad prism file for Figure 6
