# Supplementary material for: Quantification of clinically applicable stimulation parameters for precision near-organ neuromodulation of human splenic nerves
Source: Commun Biol. 2020 Oct 16;3:577. doi: 10.1038/s42003-020-01299-0 (PMC7568572; doi:10.1038/s42003-020-01299-0)
Supplement: Supplementary file 8 — Reporting Summary [file 42003_2020_1299_MOESM8_ESM.pdf]

nature research

COMMSBIO-20-0318B
Corresponding author(s): Dr. Isha Gupta
Last updated by author(s): Aug 30, 2020

Reporting Summary

Nature Research wishes to improve the reproducibility of the work that we publish. This form provides structure for consistency and transparency in reporting. For further information on Nature Research policies, see Authors & Referees and the Editorial Policy Checklist .

Statistics

For all statistical analyses, confirm that the following items are present in the figure legend, table legend, main text, or Methods section.

n/a	Confirmed	
☐	☒	The exact sample size (*n*) for each experimental group/condition, given as a discrete number and unit of measurement
☐	☒	A statement on whether measurements were taken from distinct samples or whether the same sample was measured repeatedly
☐	☒	The statistical test(s) used AND whether they are one- or two-sided<br>*Only common tests should be described solely by name; describe more complex techniques in the Methods section.*
☒	☐	A description of all covariates tested
☐	☒	A description of any assumptions or corrections, such as tests of normality and adjustment for multiple comparisons
☐	☒	A full description of the statistical parameters including central tendency (e.g. means) or other basic estimates (e.g. regression coefficient) AND variation (e.g. standard deviation) or associated estimates of uncertainty (e.g. confidence intervals)
☒	☐	For null hypothesis testing, the test statistic (e.g. *F*, *t*, *r*) with confidence intervals, effect sizes, degrees of freedom and *P* value noted<br>*Give P values as exact values whenever suitable.*
☒	☐	For Bayesian analysis, information on the choice of priors and Markov chain Monte Carlo settings
☒	☐	For hierarchical and complex designs, identification of the appropriate level for tests and full reporting of outcomes
☒	☐	Estimates of effect sizes (e.g. Cohen's *d*, Pearson's *r*), indicating how they were calculated

Our web collection on statistics for biologists contains articles on many of the points above.

Software and code

Policy information about availability of computer code

Data collection	Computational modeling was performed using Sim4Life platform with our external collaborator at IT'IS Foundation, Zurich.<br>Electrophysiological data was gathered in-house using data acquisition system 1401, Spike2, Labchart.<br>Histomorphometric quantification was performed using NDP viewer (Hamamatsu Photonics)
Data analysis	Python scripts was also used to analyse data.<br>Electrophysiological data was analysed using Graphpad and Microsoft Excel.

For manuscripts utilizing custom algorithms or software that are central to the research but not yet described in published literature, software must be made available to editors/reviewers. We strongly encourage code deposition in a community repository (e.g. GitHub). See the Nature Research guidelines for submitting code & software for further information.

Data

Policy information about availability of data

All manuscripts must include a data availability statement. This statement should provide the following information, where applicable:
- Accession codes, unique identifiers, or web links for publicly available datasets
- A list of figures that have associated raw data
- A description of any restrictions on data availability

Source data for figure(s) 2, 5 and 6 are provided with the paper. Any other dataset is available from the corresponding author upon reasonable request.

Field-specific reporting

Please select the one below that is the best fit for your research. If you are not sure, read the appropriate sections before making your selection.

Life sciences study design

All studies must disclose on these points even when the disclosure is negative.

Sample size	The sample size of the study was determined based on requirements of the regulatory submissions for therapy and for building statistics for stimulation parameters . A set of N=9 is presented for electrophysiology from human data.
Data exclusions	No data excluded
Replication	Every organ donor patient tissue was considered as a study and the findings were replicated from sample to sample. No data point was excluded from the study. The findings were also comparable to the computational modeling studies presented in the manuscript.
Randomization	Not relevant to study
Blinding	Not relevant to study

Reporting for specific materials, systems and methods

We require information from authors about some types of materials, experimental systems and methods used in many studies. Here, indicate whether each material, system or method listed is relevant to your study. If you are not sure if a list item applies to your research, read the appropriate section before selecting a response.

Materials & experimental systems

n/a	Involved in the study	
☒	☐	Antibodies
☒	☐	Eukaryotic cell lines
☒	☐	Palaeontology
☒	☐	Animals and other organisms
☐	☒	Human research participants
☒	☐	Clinical data

Methods

n/a	Involved in the study	
☒	☐	ChIP-seq
☒	☐	Flow cytometry
☒	☐	MRI-based neuroimaging

Human research participants

Policy information about studies involving human research participants

Population characteristics	Age: 40-63, Sex: M/F, BMI: 22-32
Recruitment	Tissues were obtained from freshly explanted human splenic neurovascular bundle obtained from organ transplant donors after ethical approval and informed consent from the donor families.
Ethics oversight	GlaxoSmithKline, Galvani Bioelectronics, Addenbrookes Hospital, Cambridge (UK Human Tissue Authority Code of Practice and Human Tissue Act 2004)

Note that full information on the approval of the study protocol must also be provided in the manuscript.
